# Gallic acid pretreatment mitigates parathyroid ischemia–reperfusion injury through signaling pathway modulation

**DOI:** 10.1038/s41598-024-63470-5

**Published:** 2024-06-05

**Authors:** Nianqiu Liu, Hongmin Liang, Yuan Hong, Xiaokai Lu, Xin Jin, Yuting Li, Shiying Tang, Yihang Li, Weihan Cao

**Affiliations:** 1 Departments of Breast Surgery, Yunnan Cancer Center, The Third Affiliated Hospital of Kunming Medical University, Kunming, 650000 Yunnan People’s Republic of China; 2 Department of Ultrasound, The First Affiliated Hospital of Kunming Medical University, 295 Xichang Road, Kunming, 650000 Yunnan People’s Republic of China; 3 Departments of Laboratory, Yunnan Cancer Center, The Third Affiliated Hospital of Kunming Medical University, Kunming, 650000 Yunnan People’s Republic of China; 4 Departments of Ultrasound, Yunnan Cancer Center, The Third Affiliated Hospital of Kunming Medical University, Kunming, 650000 Yunnan People’s Republic of China

**Keywords:** Gallic acid, Hypoparathyroidism, Ischemia–reperfusion injury, Mechanism, Parathyroid diseases, Thyroid diseases

## Abstract

Thyroid surgery often results in ischemia–reperfusion injury (IRI) to the parathyroid glands, yet the mechanisms underlying this and how to ameliorate IRI remain incompletely explored. Our study identifies a polyphenolic herbal extract—gallic acid (GA)—with antioxidative properties against IRI. Through flow cytometry and CCK8 assays, we investigate the protective effects of GA pretreatment on a parathyroid IRI model and decode its potential mechanisms via RNA-seq and bioinformatics analysis. Results reveal increased apoptosis, pronounced G1 phase arrest, and significantly reduced cell proliferation in the hypoxia/reoxygenation group compared to the hypoxia group, which GA pretreatment mitigates. RNA-seq and bioinformatics analysis indicate GA’s modulation of various signaling pathways, including IL-17, AMPK, MAPK, transient receptor potential channels, cAMP, and Rap1. In summary, GA pretreatment demonstrates potential in protecting parathyroid cells from IRI by influencing various genes and signaling pathways. These findings offer a promising therapeutic strategy for hypoparathyroidism treatment.

## Introduction

Hypoparathyroidism is characterized by reduced blood calcium and elevated blood phosphorus due to inadequate parathyroid hormone secretion^1^. The occurrence of hypoparathyroidism is associated with an increased risk of developing cardiovascular disease and, in severe cases, can contribute to the development of heart failure^2^. Moreover, hypoparathyroidism can lead to renal insufficiency and kidney stone formation^3^. Primary causes of hypoparathyroidism include truncation or damage to the blood vessels that supply the parathyroid branches during total thyroidectomy and inadvertent removal of parathyroid glands^4,5^. However, with advancements in surgical techniques and implementation of preoperative or intraoperative parathyroid positioning^6^, the occurrence of improper parathyroid removal has become exceedingly rare. Nevertheless, the origin and branching of the vessels supplying the parathyroid glands are often intricate and present huge variability^7^. Consequently, even when parathyroid glands are intact in situ, vascular damage may occur from clamping or thermal injury during surgical procedures, which can lead to ischemia–reperfusion injury (IRI) of the glands.

IRI tends to occur in highly perfused organs and can result in conditions such as acute kidney injury, myocardial infarction, ischemic stroke and liver failure^8^. The abrupt interruption of blood flow induces ischemia, causing damage to metabolically robust tissues. Subsequent restoration of blood flow can elicit additional cellular damage, primarily through oxygen radical-mediated injury and inflammatory cascade responses, resulting in damage to vascular endothelial cells and organ function^9^. Therefore, it is critical to improve the understanding of IRI and to develop novel therapeutic strategies. One approach is through the regulation of genes^8^. Several previous studies have shown that BNIP3, HIF-1a and the Hippo pathway are all capable of being mechanisms to ameliorate IRI^10–13^.

Using pharmacological agents to alleviate IRI has emerged as a promising clinical treatment approach. Gallic acid (GA), also known as 3,4,5-trihydroxybenzoic acid, is a plant-derived polyphenolic compound with low molecular weight^14^. A previous study showed that GA exerts antioxidant properties by inhibiting cell apoptosis and suppressing the progression of inflammatory processes^14^. In addition, numerous studies have consistently demonstrated the protective role of GA in organs that are likely to suffer from IRI, such as the heart, brain and kidney. GA can ameliorate left ventricular dysfunction and hypertrophy, which are associated with diabetes, and reduce the ischemia–reperfusion-induced myocardial infarction area^15,16^. It can also mitigate oxidative stress injury caused by renal ischemia-reperfusion^17^, elevate antioxidant levels in brain tissue and reduce cerebral infarction attributed to cerebral IRI^18^. After pretreating patients with cyclophosphamide, GA can diminish inflammatory and oxidative stress injury to the kidneys in IRI induced by cyclophosphamide^19^. Collectively, these findings indicated that GA can mitigate IRI.

Notably, to the best of our knowledge, the potential benefits of GA pretreatment in parathyroid IRI have not been thoroughly explored. Therefore, the aim of the present study was to evaluate the protective effect of GA on parathyroid cells following IRI and to investigate the underlying mechanism.

## Results

### IRI reduces cell proliferation by inducing cell apoptosis and inducing cell cycle arrest

**Figure 1:**
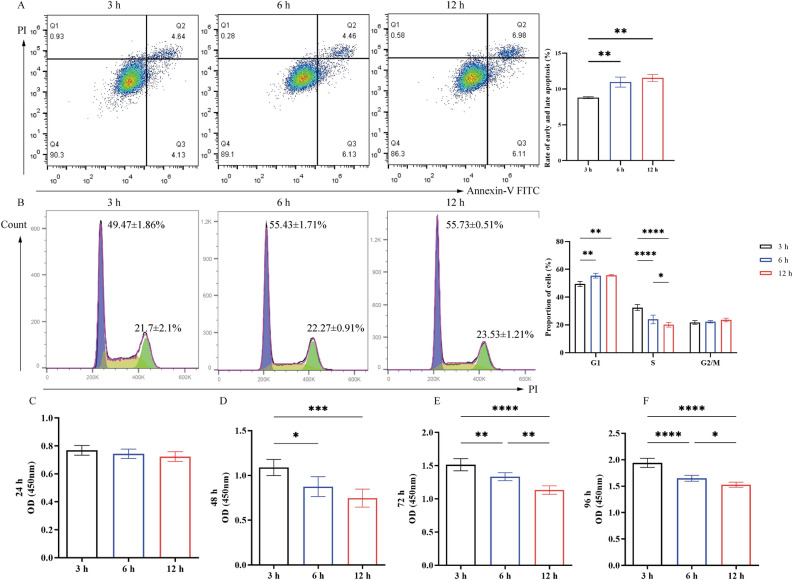
Changes in (**A**) early and late apoptosis, (**B**) cell cycle progression and (**C**) proliferative alterations in parathyroid cells during a 24-h period following incubation in 1% oxygen for 3, 6 or 12 h, (**D**) proliferative alterations in parathyroid cells during a 48-h period following incubation in 1% oxygen for 3, 6 or 12 h, (**E**) proliferative alterations in parathyroid cells during a 72-h period following incubation in 1% oxygen for 3, 6 or 12 h, (**F**) proliferative alterations in parathyroid cells during a 96-h period following incubation in 1% oxygen for 3, 6 or 12 h. ^****^P < 0.0001, ^**^P < 0.01, ^*^P < 0.05.

The hypoxia (Hypo) group was generated by culturing primary parathyroid cells in 1% O_2_ for 3, 6 or 12 h, after which, apoptosis, cell cycle progression and cell proliferation were assessed. The results showed that the early and late apoptotic rate gradually increased (**P < 0.01) with increasing hypoxic incubation time (3 h (8.79 ± 0.13%) vs. 6 h (10.97 ± 0.70%) vs. 12 h (11.55 ± 0.49%)) (Fig. 1A). Furthermore, halted cell cycle transition from G_1_ phase to S phase was observed (3 h (G1-49.47 ± 1.86%, S-32.5 ± 2.1%, G2/M-21.7 ± 1.35%) vs. 6 h (G1-55.43 ± 1.71%, S-23.97 ± 3.15%, G2/M-22.27 ± 0.91%) vs. 12 h (G1-55.73 ± 0.51%, S-20.07 ± 1.67%, G2/M-23.53 ± 1.21%) , ****P < 0.0001, **P < 0.01, *P < 0.05) (Fig. 1B), as well as reduced cell proliferation (3 h vs. 6 h vs. 12 h, at 24 h (3 h-0.78 ± 0.03, 6 h-0.74 ± 0.03, 12 h-0.73 ± 0.04), 48 h (3 h-1.09 ± 0.09, 6 h-0.88 ± 0.11, 12 h-0.75 ± 0.10), 72 h (3 h-1.52 ± 0.09, 6 h-1.34 ± 0.06, 12 h-1.13 ± 0.06) and 96 h (3 h-1.94 ± 0.09, 6 h-1.65 ± 0.05, 12 h-1.53 ± 0.05), ****P < 0.0001, ***P < 0.001, **P < 0.01, *P < 0.05) (Fig. 1C–F). Notably, no significant difference in terms of apoptosis and proportion of cells in G_1_ phase was observed between cells cultured for 6 and 12 h, which suggested that the hypoxia-induced apoptosis of parathyroid cells may have plateaued at 6 h.

**Figure 2:**
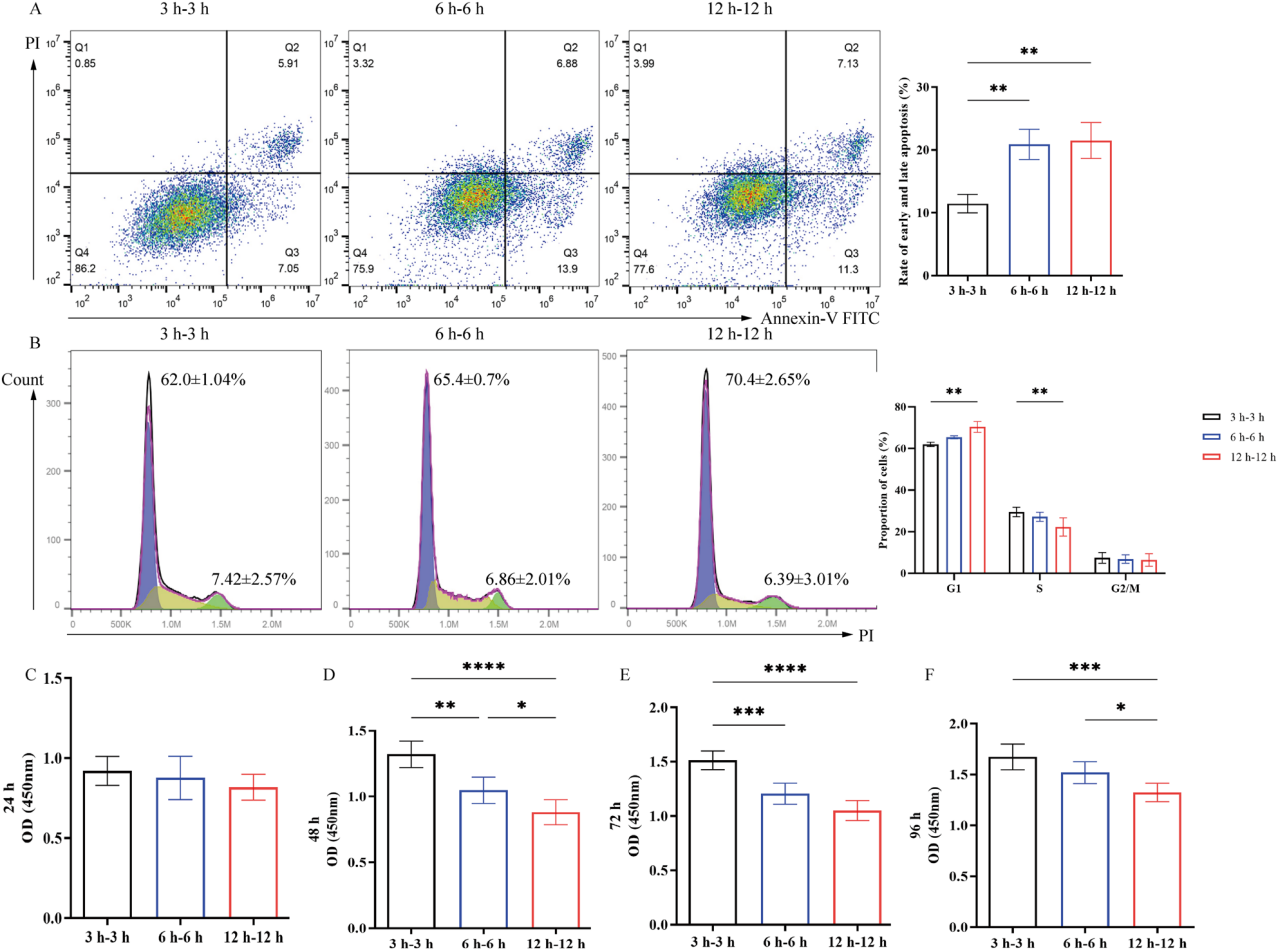
Changes in (**A**) early and late apoptosis, (**B**) cell cycle progression and (**C**) proliferative alterations in parathyroid cells during a 24-h period following incubation in 1% oxygen for 3, 6 or 12 h, followed by incubation with 21% O_2_ for 3, 6 or 12 h, respectively, (**D**) proliferative alterations in parathyroid cells during a 48-h period following incubation in 1% oxygen for 3, 6 or 12 h, followed by incubation with 21% O_2_ for 3, 6 or 12 h, respectively, (**E**) proliferative alterations in parathyroid cells during a 72-h period following incubation in 1% oxygen for 3, 6 or 12 h, followed by incubation with 21% O_2_ for 3, 6 or 12 h, respectively, (**F**) proliferative alterations in parathyroid cells during a 96-h period following incubation in 1% oxygen for 3, 6 or 12 h, followed by incubation with 21% O_2_ for 3, 6 or 12 h, respectively. ^****^P < 0.0001, ^***^P < 0.001, ^**^P < 0.01, ^*^P < 0.05.

Subsequently, an IRI model was established by resupplying 21% O_2_ to parathyroid cells that were cultured with 1% O_2_ for 3, 6 or 12 h for another 3, 6 or 12 h, respectively. The results demonstrated a gradual increase (**P < 0.01) in apoptosis (3 h-3 h (11.45 ± 1.47%) vs. 6 h-6 h (20.88 ± 2.40%) vs. 12 h-12 h (21.52 ± 2.85%)) (Fig. 2A) and cell cycle arrest in G_1_ phase (3 h-3 h (G1-62.00 ± 1.04%, S-29.50 ± 2.26%, G2/M-7.42 ± 2.57%) vs. 6 h-6 h (G1-65.4 ± 0.70%, S-27.17 ± 2.22%, G2/M-6.86 ± 2.01%) vs. 12 h-12 h (G1-70.4 ± 2.65%, S-22.3 ± 4.42%, G2/M-6.39 ± 3.01%), **P < 0.01) (Fig. 2B), as well as reduced cell proliferation (3 h-3 h vs. 6 h-6 h vs. 12 h-12 h, at 24 h (3 h-3 h-0.92 ± 0.09, 6 h-6 h-0.88 ± 0.14, 12 h-12 h-0.82 ± 0.08), 48 h (3 h-3 h-1.32 ± 0.10, 6 h-6 h-1.05 ± 0.10, 12 h-12 h-0.88 ± 0.09), 72 h (3 h-3 h-1.51 ± 0.09, 6 h-6 h-1.21 ± 0.10, 12 h-12 h-1.05 ± 0.09) and 96 h (3 h-3 h-1.67 ± 0.13, 6 h-6 h-1.52 ± 0.11, 12 h-12 h-1.33 ± 0.09), ****P < 0.0001, ***P < 0.001, **P < 0.01, *P < 0.05) (Fig. 2C–F). However, no significant difference in apoptotic rate was observed between the 6 h-6 h and 12 h-12 h groups.

**Figure 3:**
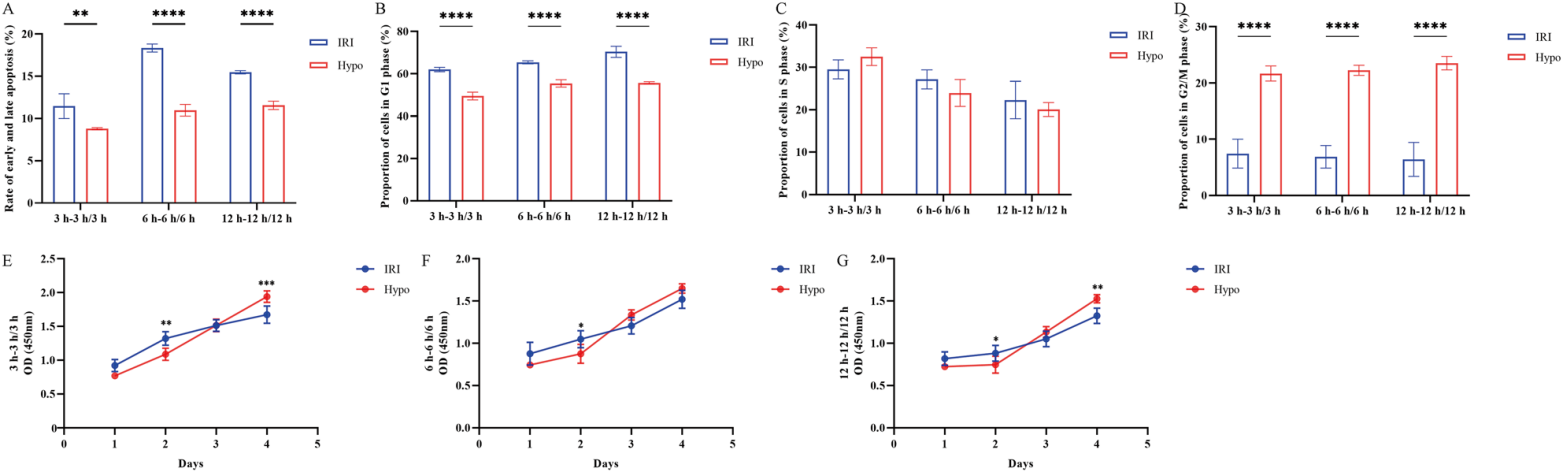
Comparisons of (**A**) early and late apoptotic rate, (**B**) proportion of cells in G1 phase, (**C**) proportion of cells in S phase and (**D**) proportion of cells in G2/m phase, and (**E**) proliferative alterations in parathyroid cells from 24 to 96 h between the IRI group and the Hypo group at 3 h-3 h/3 h, (**F**) proliferative alterations in parathyroid cells from 24 to 96 h between the IRI group and the Hypo group at 6 h-6 h/6 h, (**G**) proliferative alterations in parathyroid cells from 24 to 96 h between the IRI group and the Hypo group at 12 h-12 h/12 h. The original flow cytometry plots for the data shown in A are in Figs. 1A and 2A, and those for the data shown in B–D are in Figs. 1B and 2B. ^****^P < 0.0001, ^***^P < 0.001, ^**^P < 0.01, ^*^P < 0.05. *Hypo* hypoxia; *IRI* ischemia–reperfusion injury.

The present results revealed that apoptosis was more pronounced in the IRI groups compared with that in the Hypo groups (3 h-3 h vs. 3 h, 6 h-6 h vs. 6 h, 12 h-12 h, ****P < 0.0001, **P < 0.01) (Fig. 3A), as was the cell cycle arrest in G_1_ phase (3 h-3 h vs. 3 h, 6 h-6 h vs. 6 h, 12 h-12 h, ****P < 0.0001) (Fig. 3B-D). In addition, the reduction in cell proliferation was more pronounced after 72 h in the IRI group compared with that in the Hypo group (***P < 0.001, **P < 0.01, *P < 0.05) (Fig. 3E,G), although not statistically significant (Fig. 3F). These findings indicated that IRI could induce further damage to parathyroid cells compared with hypoxia and identified the time-point of 6 h-6 h for further experiments.

### GA partially alleviates IRI-induced damage

**Figure 4:**
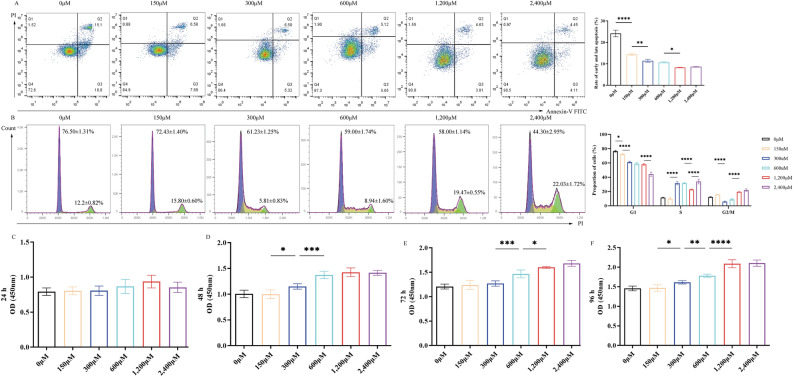
Changes in (**A**) early and late apoptosis, (**B**) cell cycle progression, (**C**) proliferative alterations in parathyroid cells during a 24-h period, (**D**) proliferative alterations in parathyroid cells during a 48-h period, (**E**) proliferative alterations in parathyroid cells during a 72-h period, (**F**) proliferative alterations in parathyroid cells during a 96-h period, pretreated with different concentrations of gallic acid for 24 h before being cultured in a hypoxic environment for 6 h and an oxygenated environment for an additional 6 h. ^****^P < 0.0001, ^***^P < 0.001, ^**^P < 0.01, ^*^P < 0.05.

**Figure 5:**
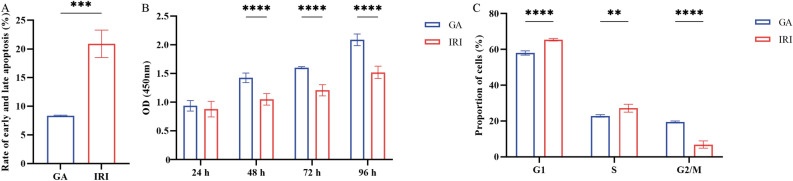
Comparisons of (**A**) apoptosis, (**B**) proliferation and (**C**) cell cycle progression between parathyroid cells that were pretreated with GA at 1200 μM for 24 h, or non-treated cells following exposure to 6 h-6 h IRI. The original flow cytometry plots for the data shown in (**A**) are in Figs. 2A and 4A, and those for the data shown in (C) are in Figs. 2B and 4B. ^****^P < 0.0001, ^***^P < 0.001, ^**^P < 0.01. *OD* optical density; *GA* gallic acid; *IRI* ischemia–reperfusion injury.

Based on the aforementioned results, the 6 h-6 h time point was used to investigate whether GA could protect cells from IRI. Parathyroid cells were pretreated with six concentrations of GA for 24 h before being exposed to a hypoxic environment. The results showed that the apoptotic rate gradually decreased (0 μM-24.2 ± 1.54%, 150 μM-14.44 ± 0.58%, 300 μM-11.43 ± 0.70%, 600 μM-10.69 ± 0.23%, 1200 μM-8.34 ± 0.10%, 2400 μM-8.69 ± 0.19%, ****P < 0.0001, **P < 0.01, *P < 0.05) with increasing drug concentrations (Fig. 4A), and the proportion of cells at G_1_ phase gradually decreased (0 μM-76.5 ± 1.31%, 150 μM-72.43 ± 1.40%, 300 μM-61.23 ± 1.25%, 600 μM-59.00 ± 1.74%, 1200 μM-8.34 ± 0.10%, 2400 μM-8.69 ± 0.19%, ****P < 0.0001, *P < 0.05), whereas the proportion of cells that entered S phase gradually increased (Fig. 4B). Furthermore, cell proliferation exhibited an ascending trend (at 48 h (0 μM-1.01 ± 0.07, 150 μM-1.00 ± 0.08, 300 μM-1.15 ± 0.05, 600 μM-1.38 ± 0.07, 1200 μM-1.43 ± 0.08, 2400 μM-1.42 ± 0.05), 72 h (0 μM-1.21 ± 0.05, 150 μM-1.24 ± 0.09, 300 μM-1.27 ± 0.06, 600 μM-1.47 ± 0.08, 1200 μM-1.60 ± 0.02, 2400 μM-1.68 ± 0.06) and 96 h (0 μM-1.46 ± 0.06, 150 μM-1.47 ± 0.08, 300 μM-1.61 ± 0.04, 600 μM-1.78 ± 0.04, 1200 μM-2.01 ± 0.10, 2400 μM-2.10 ± 0.08), ****P < 0.0001, ***P < 0.001, **P < 0.01, *P < 0.05)in response to increasing concentrations of GA (Fig. 4D–F). Comparing the cells treated with different concentrations of GA, cells treated with 1200 μM exhibited the most significant changes and were thus chosen for subsequent experiments. The results revealed that pretreatment with 1200 μM GA significantly inhibited apoptosis (GA vs. IRI, ***P < 0.001) (Fig. 5A), enhanced cell proliferation (GA vs. IRI, at 48 h, 72 h and 96 h,****P < 0.0001) (Fig. 5B) and restored cell cycle progression (GA vs. IRI, ****P < 0.0001, **P < 0.01) (Fig. 5C).

### RNA-seq and bioinformatics analysis show the potential mechanism of GA in IRI

**Figure 6:**
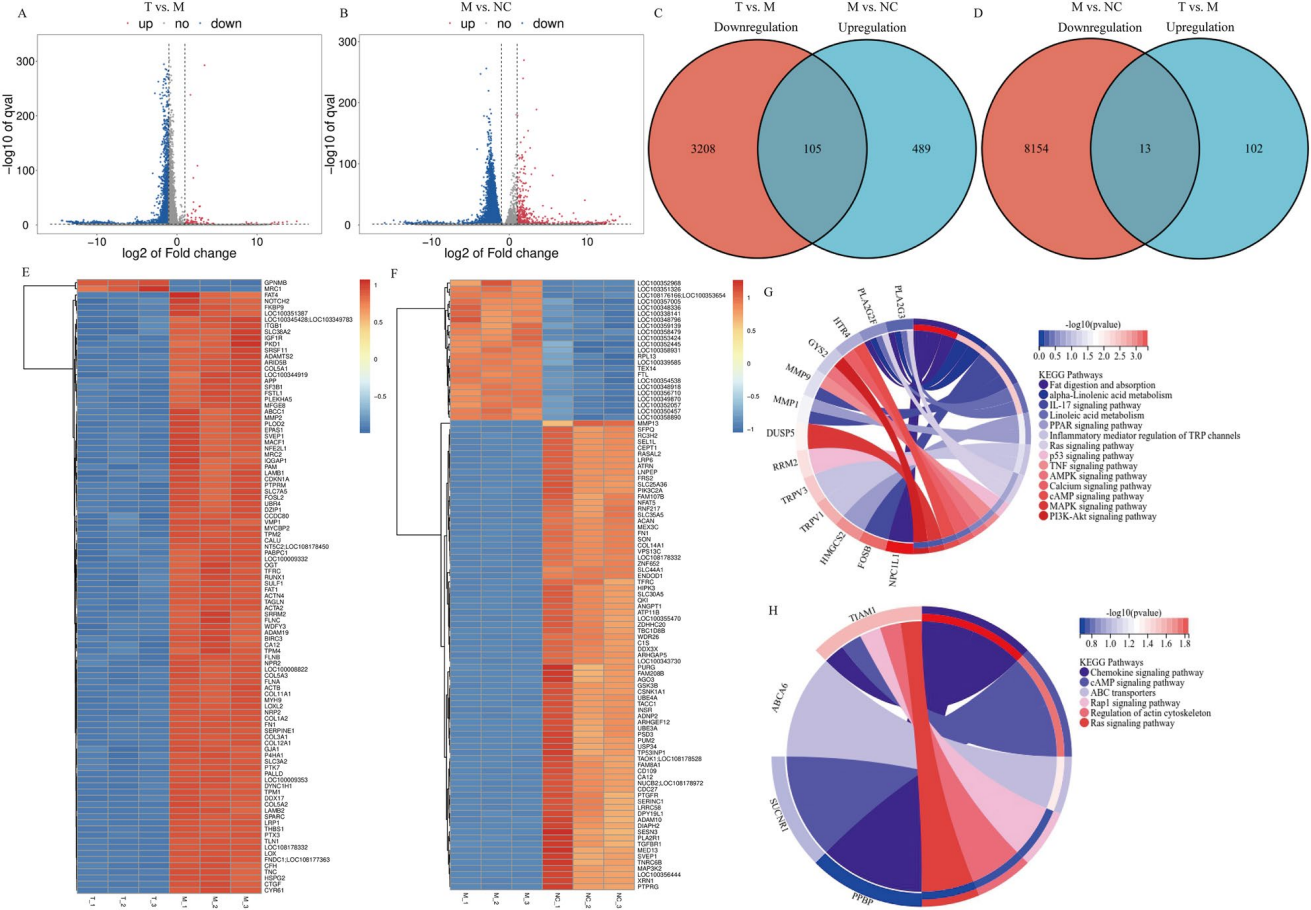
RNA-sequencing analysis of mRNA expression changes. (**A** and **E**) Differentially expressed genes in the T group compared with the M group, and (**B** and **F**) differentially expressed genes in the M group compared with the NC group. Intersection analysis of (**C**) downregulated genes in the T vs. M comparison and upregulated genes in the of M vs. NC comparison, and (**D**) upregulated genes in the T vs. M comparison and the downregulated genes in the M vs. NC comparison. KEGG enrichment analysis of (**G**) genes with elevated expression in the M group and reduced expression in the T group relative to the NC group, and (**H**) genes with reduced expression in the M group and elevated expression in the T group relative to the NC group. *KEGG* Kyoto encyclopedia of genes and genomes; *M* model; *NC*, negative control; *T* treatment.

RNA-seq was performed to investigate the mechanism underlying the effects of GA on IRI. The cells were divided into the following groups: negative control (NC) group, consisting of parathyroid primary cells; model (M) group, consisting of cells that underwent IRI (6 h-6 h); treatment (T) group, consisting of cells that were pretreated with 1200 μM GA and underwent IRI (6 h-6 h). The differential expression levels of genes were analyzed, and |log_2_ (fold change)|> 1 and *P* < 0.05 were the cutoff for significantly altered genes. The results indicated that 3315 genes were significantly downregulated and 117 genes were significantly upregulated in the treatment group compared with in the model group, and 8170 genes were significantly downregulated and 596 genes were significantly upregulated in the model group compared with in the NC group (Fig. 6A,B,E,F). Further intersection analysis of the downregulated genes in the treatment versus model comparison and the upregulated genes in the model versus NC comparison (Fig. 6C), as well as the upregulated genes in the treatment versus model comparison and the downregulated genes in the model versus NC comparison (Fig. 6D) showed a total of 105 and 13 shared genes, respectively. Subsequently, the significantly enriched KEGG signaling pathways were determined, such as ‘Fat digestion and absorption’, ‘alpha-Linolenic acid metabolism’, ‘IL-17 signaling pathway’, ‘Limoleic acid metabolism’, ‘PPAR signaling pathway’, ‘Inflammatory mediator regulation of TRP channels’, ‘Ras signaling pathway’, ‘p53 signaling pathway’, ‘TNF signaling pathway’, ‘AMPK signaling pathway’, ‘Calcium signaling pathway’, ‘cAMP signaling pathway’, ‘MAPK signaling pathway’, ‘PI3K-Akt signaling pathway’, ‘Chemokine signaling pathway’, ‘ABC transporters Rap1 signaling pathway’, and ‘Regulation of actin cytoskeleton’. A total of 13 genes (Fig. 6G), including PLA2G3, PLA2G2F, HTR4, GYS2, MMP1, MMP9, DUSP5, RRM2, TRPV3, TRPV1, HMGCS2, FOSB and NPC1L1 were upregulated in the M group and downregulated in the T group, and four genes (Fig. 6H), including TIAM1, ABCA6, SUCNR1 and PPBP were downregulated in the M group and upregulated in the T group.

## Discussion

During thyroid surgery, the parathyroid glands are susceptible to damage, which can result in IRI. IRI can further induce hypocalcemia and other clinical symptoms, such as convulsions. In severe cases, it may severely affect vital organs, such as the heart and kidneys. Consequently, careful consideration must be given to minimize the impact of IRI^1,8^. The present study established an Hypo model using primary parathyroid cells and an ischemia–reperfusion model to explore the effect of GA on IRI. The results showed that when applied prior to the occurrence of ischemia–reperfusion, GA significantly inhibited IRI-induced apoptosis, restored the normal cell cycle and promoted cell proliferation, thereby confirming the protective effect of GA on parathyroid cells.

GA is a low molecular weight tri-phenolic compound that has potent free radical-scavenging properties, and notable anti-inflammatory and anti-oxidative stress effects^20^. It is well known that IRI triggers oxidative stress injury by producing reactive oxygen species, which further induce apoptosis and the secretion of inflammatory factors^21,22^. Previous studies^20–22^ have indicated that GA may possess the potential to ameliorate cellular damage caused by ischemia–reperfusion, which was verified in the present study.

To explore the mechanism of GA in IRI, the present study conducted an RNA-seq analysis and a comprehensive literature review, which revealed that GA likely exerts its anti-oxidative stress effect through regulating the expression or function of MMP1, MMP9, DUSP5, GYS2, TRPV3, TRPV1, FOSB, TIAM1, SUCNR1 and PPBP^23–36^. According to the KEGG signaling pathway enrichment analysis, MMP1, MMP9 and FOSB were found to be enriched in the ‘IL-17 signaling pathway’. When IRI occurs, cytokines, chemokines and MMPs are released to trigger an inflammatory response and activate the IL-17 signaling pathway^37^, which in turn exacerbates tissue damage. Previous studies have revealed that inhibiting the IL-17 pathway can significantly alleviate IRI in the heart^38–40^. MMP1 and MMP9 belong to the MMP family, and downregulating their expression has been shown to reduce IRI in the heart and brain^23–26^. FOSB serves as a component of AP-1 and serves a crucial role in upregulating the activity of inflammatory macrophages during IRI^27,28^. The present findings indicated that the expression levels of MMP1, MMP9 and FOSB were significantly increased during hypoxia-reoxygenation injury and ameliorated following pretreatment with GA. These results suggested that GA may effectively mitigate ischemia–reperfusion-related inflammation by downregulating the expression of MMP1, MMP9 and FOSB. Previous studies showed that GA can activate the PPAR signaling pathway by downregulating MMP1^41,42^. Additionally, GA can inhibit activation of the TNF signaling pathway, which leads to the downregulation of MMP9 expression^43,44^. Collectively, these results demonstrated that the protective effects of GA on parathyroid cells undergoing IRI may be via regulating the expression levels of MMP1, MMP9 and FOSB.

The AMPK signaling pathway has a pivotal role in IRI as a central regulatory pathway. As a cellular energy sensor, AMPK is activated in response to substantial depletion of cellular energy. Activation of the AMPK pathway protects cells from IRI by initiating catabolism to restore ATP production, while simultaneously inhibiting anabolism to prevent further ATP depletion during ischemia-reperfusion^45^. Additionally, the AMPK pathway enhances the antioxidant activity of Nrf2 and reduces oxidative stress^45,46^. Conversely, the phosphorylation of GYS2 at Ser7 results in inactivation of GYS2 when the AMPK signaling pathway is active^29^. While the activation of the PI3K/AKT signaling pathway has been shown to mitigate IRI^47,48^, it paradoxically results in the upregulation of GYS2 expression^49^. This outcome seems contradictory to the experimental aforementioned findings. The present results demonstrated that GA alleviated IRI in parathyroid cells by downregulating GYS2 and activating the AMPK signaling pathway. However, further studies are required to determine the association among GA, GYS2 and the PI3K/AKT signaling pathway.

DUSP5 is an inducible bispecific kinase with three binding sites (Thr321, Ser346 and Ser376), which serves a crucial role in dephosphorylating ERK, JNK and p38, which are key signaling molecules within the MAPK signaling pathway. This process enables DUSP5 to regulate the function of MAPK^30,31^. Notably, when the MAPK/ERK and MAPK/p38 signaling pathways are inhibited, damage caused by ischemia–reperfusion is alleviated^50,51^. Similarly, the present results showed that GA could ameliorate IRI through regulating the DUSP5-mediated MAPK signaling pathway. However, further validation is necessary to determine the precise mechanism underlying this phenotype.

Transient receptor potential (TRP) channels have a pivotal role in mediating the release of cytokines in inflammation and oxidative stress injury^32,33^. Among these channels, TRPV1 and TRPV3, which are associated with inflammation, are primarily implicated in oxidative stress-induced cellular injury. Furthermore, TRP channels have been implicated in the pathogenesis of IRI in myocardial studies^52,53^. Similarly, the present study revealed that GA can modulate TRPV1 and TRPV3 of the TRP channels, thereby alleviating IRI.

During the reperfusion phase of IRI, the secretion of pro-inflammatory factors and macrophage-related chemokines is elevated, and can be used as surrogates to predict the extent of tissue damage^54,55^. Chemokine signaling pathways are instrumental in regulating cell polarization and inflammatory responses^56^. Deficiency of TIAM1, a key regulatory protein, leads to reduced cellular chemotaxis^34^. In addition, the expression of PPBP is positively correlated with CXCL8 expression^35^. Surprisingly, in the present study, GA pretreatment resulted in the upregulation of both TIAM1 and PPBP, in other word, IRI leads to an increase in cell chemokine levels, along with elevated expression of TIAM1 and PPBP. In theory, the application of GA should mitigate the impact of IRI on cells, thereby reducing chemokine levels, as well as the expression of TIAM1 and PPBP. However, our sequencing data indicates an increase in the expression of TIAM1 and PPBP in the T group, which is a discrepancy from the literature analysis. This suggests the existence of potentially distinct regulatory mechanisms that need to be explored in future.

The cAMP signaling pathway facilitates transduction of vasoactive transmitters, regulates vascular barriers and modulates inflammatory responses. It encompasses three principal effector proteins: Protein kinase A (PKA), cyclic-nucleotide-gated ion channels, and exchange proteins directly activated by cAMP/Epacs^57^. IRI disrupts the vascular barrier, leading to increased vascular permeability and subsequent recruitment of inflammation-associated cells, which eventually promotes the inflammatory response^58,59^. Previous studies have shown that increasing cAMP/PKA expression during IRI can effectively mitigate IRI^60,61^. TIAM1 serves a crucial role in elevating activation of the cAMP signaling pathway, thereby promoting stabilization of the vascular endothelial barrier^62^. By contrast, increased signaling of SUCNR1 leads to a decrease in cAMP expression^36^. The present results showed that GA upregulated the expression levels of both TIAM1 and SUCNR1, thereby alleviating IRI in parathyroid cells. Notably, the observed change in TIAM1 expression was consistent, whereas the change in SUCNR1 expression was inconsistent with the findings of a previous study^36^. These discrepancies need to be further examined in future studies.

Activation of the Rap1 signaling pathway promotes cellular anti-inflammatory, antioxidant and anti-apoptotic functions^63^. Additionally, within the cAMP/Epac signaling axis, Rap1 activation can stabilize the vascular endothelial barrier^62^. Notably, TIAM1 is implicated in the regulation of the cAMP signaling pathway. The results of the present study demonstrated an association between increased TIAM1 expression and the Rap1 signaling pathway identified by KEGG pathway enrichment analysis. However, further experiments are required to further determine this association. It has been discovered that downregulation of the actin cytoskeleton can attenuate oxidative stress in vascular smooth muscle during ischemia-reperfusion^64^. Conversely, elevated TIAM1 activity promotes actin cytoskeleton reorganization^65^. Thus, the TIAM1-dependent actin cytoskeleton signaling axis may not serve as a regulatory pathway for GA in ameliorating IRI. Additionally, TIAM1 is a direct mediator of Rac activation within the RAS signaling pathway that can promote intercellular adhesion^66^. Cell adhesion resulting from overexpression of vascular cell adhesion molecules is one of the pathogenic factors underlying IRI^67^. Consequently, inhibiting activation of the RAS signaling pathway can reduce apoptosis and enhance endovascular proliferation^68^. However, these findings are inconsistent with the experimental results of the present study; therefore, it is worth considering that GA may exert its regulatory role through TIAM1 in the Rap1 signaling pathway.

**Figure 7:**
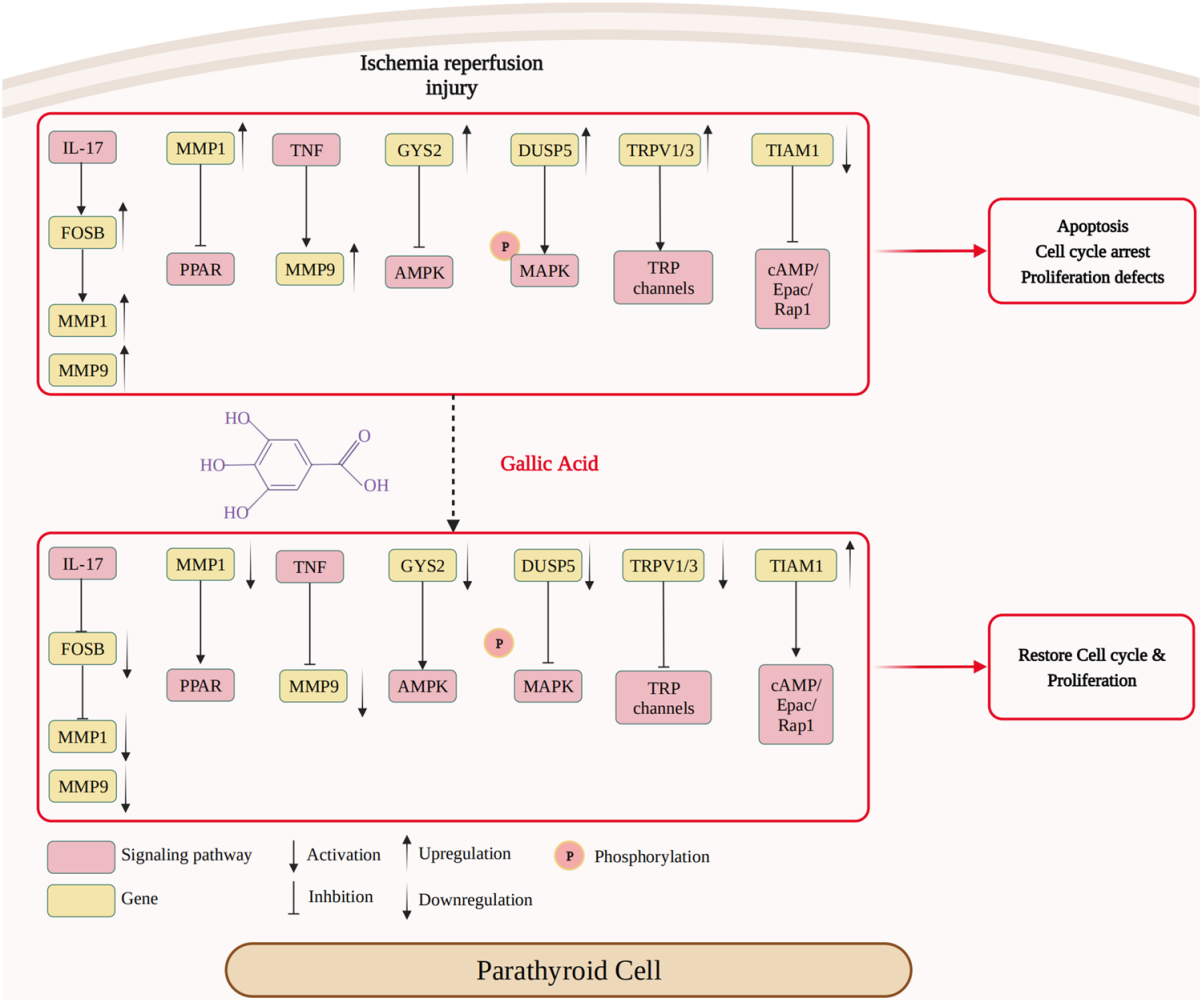
During ischemia–reperfusion injury in parathyroid cells, the expression levels of FOSB, MMP1, MMP9, GYS2, DUSP5 and TRPV1/3 were upregulated, whereas the expression of TIAM1 was downregulated. This may lead to the activation or inhibition of several corresponding signaling pathways, resulting in apoptosis, cell cycle arrest and defective proliferation of parathyroid cells. Pretreatment with gallic acid can reverse the changes in the expression of the aforementioned genes during ischemia–reperfusion injury, thus alleviating apoptosis, and restoring the normal cell cycle progression and proliferation to a certain extent.

In conclusion, the present study not only confirmed the damage induced by IRI in parathyroid cells, but also demonstrated the protective role of GA against such damage. The results showed that GA may be considered a novel therapeutic approach to manage parathyroid IRI in the clinic. Furthermore, the preliminary mechanism investigation (Fig. 7) revealed that eight genes (MMP1, MMP9, FOSB, GSY2, DUSP5, TRPV1/3 and TIAM1) that belonged to various signaling pathways may participate in the process. The identification of these genes provides a foundation for subsequent mechanistic studies and may provide information on the potential clinical application of GA under different circumstances.

## Material and methods


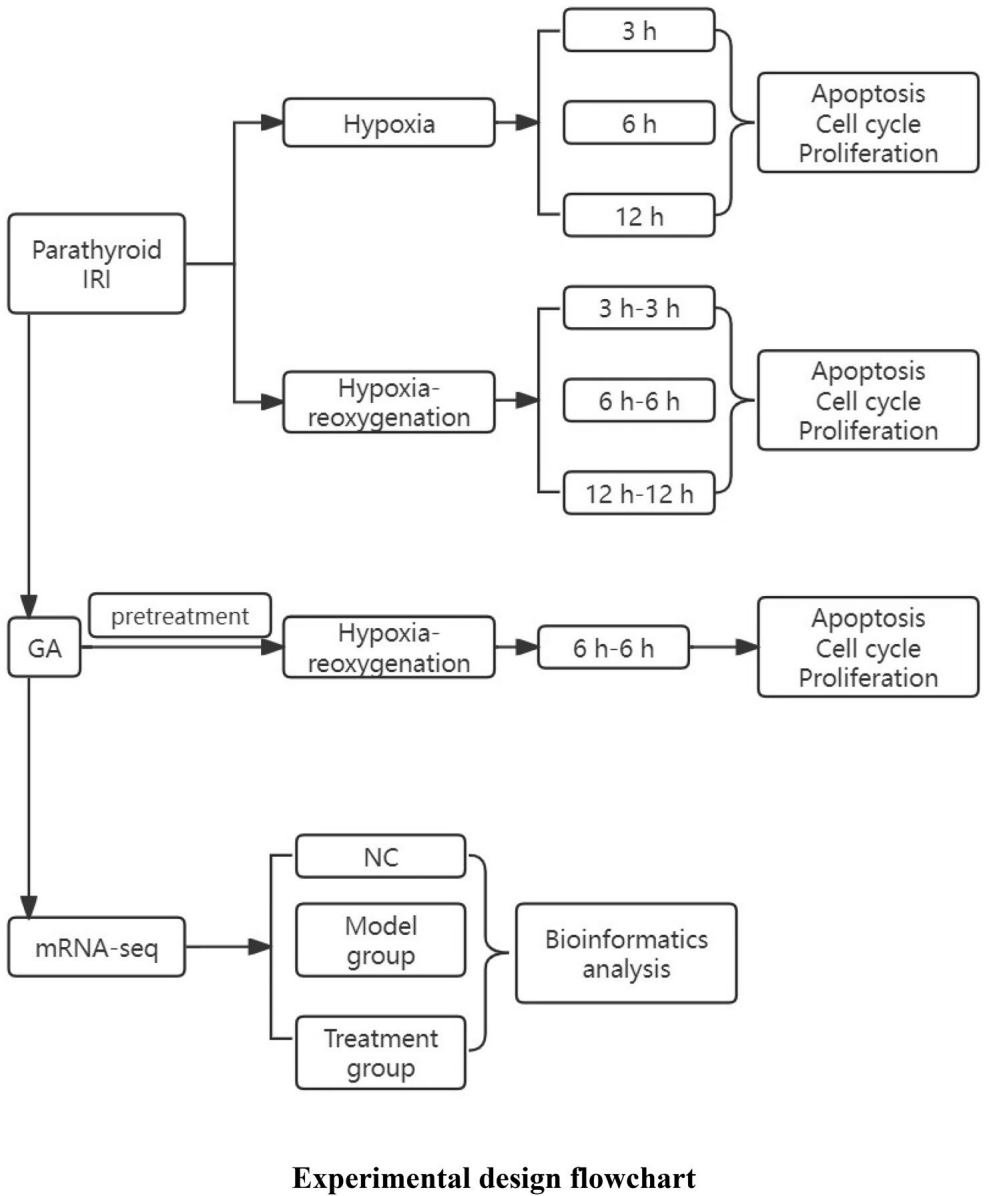


GA. GA (cat. no. S4603) was obtained from Selleck Chemicals and was dissolved in dimethyl sulfoxide (cat. No. D8370, purity > 99.9%, Solarbio) according to the manufacturer’s instructions. The official recommended starting effective concentration of GA for use in PC12 cells is 50 μM. To design the drug concentration gradients, this value was used as a reference, with preliminary experiments assessing concentrations ranging from 0 to 6000 μM (data not shown) in primary parathyroid cells. For the present study, the experimental drug concentrations used were 0, 150, 300, 600, 1200 and 2400 μM.

Primary parathyroid cell culture. The rabbit parathyroid gland primary cells (cat. no. RAB-iCell-g014) were purchased from the iCell Bioscience Inc, Shanghai, and they were cultured using the ICell Primary Epithelial Cell Culture System (cat. no. PriMed-iCell-001) under conditions of 37 °C and 5% CO2 concentration. We monitored the growth and cell structure of primary parathyroid cells on a daily basis. Additionally, we refreshed the complete culture medium every two days to ensure that the cells received the essential nutrients they required.

Cellular models. Rabbit parathyroid primary cell were obtained and were separated into the following groups: The negative control (NC) group, without any specific treatment. The hypoxia (Hypo) group, in which the progenitor cells were cultured in a 1% oxygen environment for 3, 6 or 12 h. The IRI group, in which the progenitor cells were cultured in a 1% oxygen environment for 3, 6 or 12 h, followed by reoxygenation to a normal 21% oxygen environment and continued culturing for 3, 6 or 12 h. In the treatment group, GA pretreatment was administered to the parathyroid primary cells for 24 h prior to ischemia–reperfusion.

Flow cytometric analysis of apoptosis and cell cycle progression. Cells (> 1 × 10^6^ cells/well) were grown in 6-well plates and, after adherence, were treated according to the experimental requirements. The treated cells were then digested with 0.25% trypsin, washed three times in PBS and subjected to flow cytometry using the Annexin V-FITC/PI Apoptosis detection kit (cat. no. 40302ES60; Shanghai Yeasen Biotechnology Co., Ltd.) to detect apoptosis according to the manufacturer’s protocol. For cell cycle analysis, the cells were fixed in 75% cold ethanol for 24 h at 4 °C, after ethanol fixation, centrifuge the cells at room temperature for 5 min (200 × g), carefully remove the upper ethanol layer after centrifugation, add 100 ul of PBS to gently resuspend the cells, and then centrifuge again at room temperature for 5 min (200 × g), carefully aspirate the upper PBS layer after centrifugation. and stained with 100 μl PI containing 50 μl RNaseA at room temperature to detect cell cycle progression. Flow cytometry was performed using a flow cytometer (CytoFLEX S; Beckman Coulter, Inc.) and the results were analyzed and the plots were generated using FlowJo v10 software (FlowJo, LLC).

Cell Counting Kit-8 (CCK-8) cell proliferation assay. Cells were seeded in 96-well plates (4000 cells/well), and were treated according to the experimental requirements. Subsequently, cell proliferation was assessed at 24, 48, 72 and 96 h using a CCK-8 kit (cat. no. 40203ES80; Shanghai Yeasen Biotechnology Co., Ltd.) for 4 h and the OD values at 450 nm were measured in a multifunctional enzyme plate reader. Graphs were plotted using GraphPad Prism v9 (Dotmatics).

RNA-sequencing (RNA-seq) and bioinformatics analysis. Cells were seeded in 10-cm dishes (> 1 × 10^7^ cells/well) were treated according to the experimental requirements. Subsequently, the treated cells were subjected to RNA-seq (resulting in dataset no. GSE240162), which was performed by Hangzhou LC Biotechnology Co. (project no. Is LC-P20220413-037). R v4.0.4 (https://www.r-project.org/) was used to load DESeq2^69^ (http://www.bioconductor.org/packages/release/bioc/html/DESeq2.html)/edgeR^70^ (http://www.bioconductor.org/packages/release/bioc/html/edgeR.html) for differential expression analysis, and gene expression changes (groups T vs. M and M vs. NC) were considered statistically significant if |log2 (fold change)|> 1 and *P* < 0.05. For differential expression clustering analysis Log_10_(FPKM + 1) was used for gene expression presentation, with colors ranging from blue through to white to red indicating low to high expression, respectively. Hiplot^71^ was used to plot Venn diagrams and SangerBox^72^ was used for Kyoto Encyclopedia of Genes and Genomes (KEGG) signaling pathway enrichment analysis.

### Statistical analysis

Two-way ANOVA followed by Tukey’s multiple-comparisons test was used to analyze the differences among multiple groups when two variables were being assessed. When one variable was being assessed, one-way ANOVA and Tukey’s multiple-comparisons test was performed. All statistical analyses were performed using GraphPad Prism version 9 (GraphPad Software; Dotmatics). All experiments were repeated independently at least three times and the results are shown as the mean ± SD. *P* < 0.05 was considered to indicate a statistically significant difference.

## Data Availability

The RNA-seq datasets generated and analyzed during the current study are available in the GEO repository (https://www.ncbi.nlm.nih.gov/geo/query/acc.cgi?acc=GSE240162). The other datasets used and/or analyzed during the current study are available from the corresponding author on reasonable request.

## Abbreviations

GA: Gallic acid
IRI: Ischemia–reperfusion injury
Hypo: Hypoxia
TRP: Transient receptor potential
NC: Negative control
M: Model
T: Treament
PKA: Protein kinase A
